# Special Olympics IX National Games, Healthy Athletes Program: ‘Fit Feet’

**DOI:** 10.1186/1757-1146-4-S1-P19

**Published:** 2011-05-20

**Authors:** Angela Evans, Meagan Reeve, Rebecca Rundell, Wen Qi Ng, Daina Walton

**Affiliations:** 1 School of Health Science, University of South Australia, Adelaide, SA, 5000, Australia; 2 Clinical Director, Healthy Athletes program - 'Fit Feet', Special Olympics IX National Games, Adelaide, Australia

## 

The Special Olympics IX National Games were held in Adelaide, South Australia in April 2010. Podiatrists and podiatry students were invited to participate in the healthy athletes program to assess the feet of athletes with intellectual disability. The World Health Organisation has found that people with an intellectual disability form the largest disability population in the world. Special Olympics (SO) is an international not-for-profit organisation. Globally, SO now supports 3.1 million people in 220 countries. Each year there are 30,000 SO competitions throughout the world. Affiliated with the International Olympic Committee, SO is held in the manner of all Olympic games. There are over 500,000 Australians living with an intellectual disability. Special Olympics Australia supports around 4000 athletes in over 250 sports clubs across metropolitan and rural Australia. SO Australia offers fourteen official national sports: alpine skiing, aquatics, athletics, basketball, bocce, cricket, figure skating, football (soccer), golf, gymnastics, sailing, softball, tennis and tenpin bowling. People with intellectual disability are often overrepresented in areas of preventable health problems, SO provides invaluable opportunity for identifying the frequent health care needs. Podiatry is an essential and core element of any sports and wider mobility issues.Approximately 850 participating athletes registered for the National Games in Adelaide. ‘Fit Feet’ managed to see over 400 athletes; notable as other programs have seen 10% of athletes. Screening of athletes investigated: foot and ankle problems, footwear adequacy. Referral and education were provided as required. In addition to podiatry, the athletes were also availed dental, audiology and optometry health screenings. The Australian Podiatry Association (SA), Special Olympics Australia and the University of South Australia cooperated to provide the screening program for the SO athletes. The University of South Australia acknowledged this project with a commendation at the 2010 Chancellor’s awards for community engagement. The next SO National Games will be held in Melbourne in 2014, where podiatrists and students could have the exciting and rewarding opportunity to participate in the healthy athletes program. The Special Olympics athletes’ oath is: 'Let me win. But if I cannot win, let me brave in the attempt.'

